# A Front Line on Klebsiella pneumoniae Capsular Polysaccharide Knowledge: Fourier Transform Infrared Spectroscopy as an Accurate and Fast Typing Tool

**DOI:** 10.1128/mSystems.00386-19

**Published:** 2020-03-24

**Authors:** Carla Rodrigues, Clara Sousa, João A. Lopes, Ângela Novais, Luísa Peixe

**Affiliations:** a UCIBIO/REQUIMTE, Laboratório de Microbiologia, Faculdade de Farmácia, Universidade do Porto, Porto, Portugal; b LAQV/REQUIMTE, Departamento de Ciências Químicas, Faculdade de Farmácia, Universidade do Porto, Porto, Portugal; c Research Institute for Medicines (iMed.ULisboa), Faculdade de Farmácia, Universidade de Lisboa, Lisbon, Portugal; University of California, San Diego

**Keywords:** capsular typing, strain typing, *cps* locus, biochemical data, nosocomial outbreaks

## Abstract

Genomics-based population analysis of multidrug-resistant (MDR) Klebsiella pneumoniae motivated a renewed interest on the capsule as an evolutionary and virulence marker of clinically relevant strains. Whole-genome sequencing (WGS)-based approaches have provided great insights into the genetic variability of the capsular locus, but genotypic-biochemical capsular (K)-type correlations are lacking, hindering the establishment of a reliable framework for K-type characterization and typing. To fill this gap, we combined molecular, comparative genomics, and multivariate data analysis tools with biochemical data on the capsular locus to support the usefulness of Fourier transform infrared (FT-IR) spectroscopy as a reliable K typing tool. To validate our approach, we used a representative collection of well-defined MDR K. pneumoniae lineages involved in local or nationwide epidemics in multiple countries. With this, we demonstrate a high accuracy and resolution of our FT-IR-based spectroscopy approach for K-type discrimination that is even higher than that provided by WGS. Moreover, the specific associations established between certain K types and specific K. pneumoniae lineages with high clinical relevance, together with the accuracy, simplicity, short time to result, and inexpensive features of the method, support the value of the developed FT-IR-based approach for an easy, fast, and cost-effective strain typing. This fulfills a still unmet need for tools to support real-time monitoring and control of K. pneumoniae infections. In addition, the genotypic-biochemical correlations established provide insights on sugar composition/structure of newly defined K. pneumoniae capsular types.

**IMPORTANCE** Klebsiella pneumoniae is nowadays recognized as one of the most defiant human pathogens, whose infections are increasingly more challenging to treat and control. Whole-genome sequencing (WGS) has been key for clarifying the population structure of K. pneumoniae, and it is still instrumental to provide insights into potential pathogenicity and evolutionary markers, such as the capsular locus. However, this information and WGS are still far from being accessible and translated into routine clinical microbiology laboratories as quick and cost-efficient strain diagnostic tools. Here, we propose a biochemical fingerprinting approach based on Fourier transform infrared spectroscopy (FT-IR) and multivariate data analysis tools for K. pneumoniae capsular typing that, because of its high resolution, speed, and low cost, can be an asset to provide enough information to support real-time epidemiology and infection control decisions. Besides, it provides a simple framework for phenotypic/biochemical validation of K. pneumoniae capsular diversity.

## Abstract (precis)

Klebsiella pneumoniae is nowadays recognized as one of the most defiant human pathogens, whose infections are increasingly more challenging to treat and control. Whole-genome sequencing (WGS) has been key for clarifying the population structure of K. pneumoniae, and it is still instrumental to provide insights into potential pathogenicity and evolutionary markers, such as the capsular locus. However, this information and WGS are still far from being accessible and translated into routine clinical microbiology laboratories as quick and cost-efficient strain diagnostic tools. Here, we propose a biochemical fingerprinting approach based on Fourier transform infrared spectroscopy (FT-IR) and multivariate data analysis tools for K. pneumoniae capsular typing that, because of its high resolution, speed, and low cost, can be an asset to provide enough information to support real-time epidemiology and infection control decisions. Besides, it provides a simple framework for phenotypic/biochemical validation of K. pneumoniae capsular diversity.

## INTRODUCTION

Klebsiella pneumoniae is an encapsulated bacterium nowadays recognized as one of the most challenging human pathogens due to the increasing rates of mortality and morbidity associated with severe infections (hypervirulent [HV] strains) or with high rates of infections by strains resistant to multiple antibiotics (multidrug-resistant [MDR] strains) (1, 2). The lack of efficient strain typing tools accessible to the clinical microbiology routine laboratories (in what regards simplicity, time, and cost) has been hampering the control of K. pneumoniae in clinical settings.

The capsule, an extracellular polysaccharide matrix, is one of the most striking virulence mechanisms considered essential for establishment of infection and for its protective effect against desiccation, phages, and protists predation (1). Though it renders attractive properties as a target for vaccine development, the success of immunotherapeutic approaches depends on a complete understanding of bacterial surface structures circulating in the clinical setting (3, 4). Whereas it is known that the expression of specific virulence factors and capsular (K) types (mainly K1 and K2) is related to severe infections caused by HV strains, much larger variation of K types has been described among clinical MDR strains, providing a higher resolution than multilocus sequence typing (MLST) and specific capsule-lineage associations that might be useful for typing (5–7). Variation on K antigens and also in other surface polysaccharides (such as O antigen) has been traditionally used for *Klebsiella* typing. In fact, serotyping, established as early as 1926 (8), allowed the recognition of 77 serologically distinct K types (K1 to K82) and much less diverse O types (*n* = 8, O1 to O12) among the reference strain collection, deposited at Statens Serum Institute, Copenhagen, Denmark (9, 10). The chemical composition and structure of capsular types has been clarified essentially during the 1980s for strains from the reference collection, but correlation with genomic data is recent and not always straightforward (11). The lack of practicability (it is complex and laborious) and availability (only at reference centers) of serotyping and the insufficient coverage led to its almost complete abandonment in the past decades (1).

Given the renewed interest in capsular polysaccharides, several genotypic methods have recently been proposed to revive K typing, but there are several flaws that prevent their universal application and coverage. Molecular methods to infer K type from genomic data such as restriction fragment length polymorphism (RFLP) of the *cps* locus (generating “C patterns”) or PCR targeting specific K types (e.g., K1, K2, and K57) are technically demanding, have low coverage, and/or are not suitable to detect variation in other sites of the locus (12, 13). K-type prediction based on allelic variation of *loci* (e.g., *wzi* or *wzc*) within the capsule biosynthetic pathway (*cps* locus) constitute more rapid and simple approaches (14, 15), but the characterization of the whole *cps* and *rfb* (O-antigen biosynthesis) locus by whole-genome sequencing (WGS) improved accessibility and precision, especially through user-friendly Web-based platforms, such as Kaptive (http://kaptive.holtlab.net) (7, 16). These *in silico* studies and others uncovered a series of novel *cps* loci and at least 161 presumptive phenotypically distinct capsular types (designated KL to differentiate from the reference K types) (7, 16–18). More importantly, these data revealed the usefulness of K variation as an epidemiological marker for strain subtyping, encouraging the development of reliable, fast, and high-throughput tools for K typing (7, 17, 19–21).

Fourier transform infrared (FT-IR) spectroscopy has been shown to detect surface phenotypic differences linked to a variable composition on glycan structures that form part of the O and K antigens, depending on the bacterial species (22–25). Considering that the capsule is the outermost structure of K. pneumoniae, we hypothesize that FT-IR spectroscopy is a reliable tool to detect variations on K. pneumoniae capsule composition, as observed for other capsulated bacteria (26, 27). In this study, we combined molecular genotypic, comparative genomics, and biochemical data associated with the *cps* locus and multivariate data analysis tools to assess the potential and robustness of FT-IR spectroscopy for the identification and characterization of K. pneumoniae capsular types. The high congruence established between capsular genotypic and biochemical features (adjusted Wallace coefficient, 0.966 to 1.000) opens new avenues for a more comprehensive understanding of K-type variation and evolution among MDR K. pneumoniae lineages and supports the potential of the methodology as a suitable K. pneumoniae typing tool. The significance of the results for K. pneumoniae typing and for a better understanding of host-pathogen interactions is also discussed.

## RESULTS

### Molecular genotypic characterization of K. pneumoniae K antigen.

The existing methodologies for K typing are suboptimal and there is a lack of genotype-biochemical correlation. We thus used FT-IR spectroscopy combined with molecular methods to identify known K types or predict the composition of unknown K types.

Our approach was validated on a collection of 154 well-characterized MDR K. pneumoniae isolates that had been involved in local or nationwide epidemics in different countries from Europe and South America spanning a long period of time (2002 to 2015). These isolates were selected to capture a diversity of capsular types harbored by the main diverse K. pneumoniae lineages from different clonal groups (CG) that had been involved in human clinical infections and in the expansion of extended-spectrum β-lactamases (mainly, CTX-M-15 and SHV-12) and/or carbapenemases (mainly, KPC-type, OXA-48, VIM, and NDM) (Table 1). The results will be compared with the performance of different cutting-edge genotypic K-typing methods.

**TABLE 1:** Detailed characterization of the 154 international MDR K. pneumoniae clinical isolates analyzed in this study

ST/CG (no.)	K/KL type by genotypic methods^*a*^ (no.)	O type^*b*^	FT-IR type (no.)	PFGE type(s) (no.)	Country	Yr(s) of isolation	β-Lactamase(s) conferring resistance to extended-spectrum β-lactams
ST11/CG258 (32)	K24 (12)	O1, O2	FT5 (11), FT24 (1)	Kp13 (1), Kp14 (10), Kp15 (1)	Spain, Portugal	2010–2012	DHA-1, OXA-48, KPC-3
	K27 (8)	O2	FT17	Kp17 (6), Kp18 (1), Kp19 (1)	Brazil	2012	KPC-2, CTX-M-2
	K64^*f*^ (3)	O2	FT4	Kp28	Brazil	2012	KPC-2, CTX-M-2
	KL105 (7)	O2	FT13 (5), FT23 (2)	Kp30	Portugal	2006–2013	DHA-1, DHA-6
	KL127 (2)	—^*c*^	FT16	Kp31	Brazil	2009–2012	KPC-2
ST14/CG14 (9)	K2 (3)	O1	FT14	Kp16	Portugal	2002–2003	TEM-24
	K16 (6)	O1	FT15	Kp32	Portugal	2010	SHV-55, SHV-106
ST15/CG15 (33)	K19 (6)	O1	FT10	Kp1	Portugal	2012–2015	KPC-3, CTX-M-15
	KL112 (11)	O1	FT12 (10), FT27 (1)	Kp5	Portugal	2010–2012	CTX-M-15
	K24 (13)	O1	FT5 (11), FT21 (1), FT26 (1)	Kp12	Portugal, Brazil	2006–2014	CTX-M-15, OXA-48, SHV-2
	KL110 (2)	O1	FT19	Kp21	Portugal	2011–2012	VIM-34, SHV-12, OXA-17
	KL48 (1)	O1	NI^*d*^	Kp12	Portugal	2010	SHV-12
ST17/CG17 (4)	KL112 (3)	O2, O5	FT12 (2), FT22 (1)	Kp6 (2), Kp7 (1)	Brazil, Portugal	2012	DHA-1, SHV-2, SHV-12
	(*wzi*200)^*e*^ (1)	—	NI	Kp34	Spain	2012	VIM-1
ST39/CG39 (7)	K23 (7)	O1	FT1	Kp33	Spain	2008–2010	VIM-1
ST54/— (3)	K14^*f*^ (3)	O3	FT2	Kp29	Spain	2009–2010	VIM-1
ST101/CG101 (11)	K17 (11)	O1	FT8 (8), FT20 (3)	Kp8 (6), Kp9 (2), Kp10 (2), Kp11 (1)	Romania, Brazil	2012	OXA-48, OXA-181, NDM-1, KPC-2, CTX-M-2, CTX-M-15
ST147/CG147 (15)	K64^*f*^ (15)	O2, O1	FT4	Kp26 (14), Kp27 (1)	Spain, Portugal	2006–2015	KPC-3, SHV-12, VIM-1
ST253/— (3)	K60 (3)	O1	FT3	Kp20	Spain	2009–2010	VIM-1
ST258/CG258 (16)	KL106 (14)	O2	FT6 (12), FT25 (2)	Kp24 (6), Kp2 (4), Kp25 (4)	Greece, Poland, Brazil	2007–2009	KPC-2, CTX-M-2, SHV-12
	KL107 (2)	O2	FT9	Kp2	Poland	2008–2009	KPC-3, CTX-M-3
ST336/CG17 (12)	(*wzi*150)^*e*^ (12)	—	FT24	Kp23	Portugal	2010	CTX-M-15
ST348/— (4)	K62 (4)	O1	FT11	Kp22	Portugal	2012	KPC-3, CTX-M-15
ST405/— (5)	KL151 (5)	O4	FT7	Kp3 (4), Kp4 (1)	Portugal, Spain	2012–2013	OXA-48, CTX-M-15

a Capsular (K) type defined according to Brisse et al. (14) and capsular locus (KL) type according to Wick et al. (7).

b O types defined according to Fang et al. (10).

c —, not defined.

d NI, not included.

e *wzi* allele associated with no or multiple K/KL types.

f Cross-reaction between K14 and K64, solved by sequencing of *wzy*.

### (i) Capsular assignment based on the genotypic marker *wzi*.

First, K types were inferred by sequence comparison of a discriminatory molecular marker (*wzi*) (14). Twenty-two different *wzi* alleles were identified, four of which (*wzi*89 and *wzi*200 to -202) were new and deposited at the BIGSdb-*Kp* Pasteur database (http://bigsdb.pasteur.fr/klebsiella/klebsiella.html), and described meanwhile in other studies (16). According to this database, 13 *wzi* alleles were unequivocally associated with one unique K type (positive reaction with the sera from reference K types) and/or KL type (predicted based on *cps* locus obtained by WGS data, when available) (Table 2). However, prediction of K type was not always straightforward, since (i) 6 *wzi* alleles were linked to more than one K type/KL type, (ii) 2 *wzi* alleles (*wzi*29 and *wzi*93) were linked to discordant K type/KL type, and (iii) 1 *wzi* allele (*wzi*200) has no K/KL type attributed (Table 2).

**TABLE 2:** K/KL type prediction by the different methods tested

*wzi* allele	K type^*a*^	KL type^*b*^	Epidemiological data^*c*^	FT-IR
2	K2	KL2.KL30	**KL2**	K2
14	K14	KL14	KL14	K14
16	K16	KL16.KL143	**KL16**	K16
19	K19	KL19	**KL19**	K19
24	K24	KL24.KL54.KL55	**KL24**	K24^*d*^
27	K27	KL27	KL27	K27
29	K41	KL106	KL106	KL106^*d*^
64	K14.K64	KL64	KL64	KL64
75	—^*e*^	KL105	**KL105**	KL105^*d*^
83	K23	KL23	KL23	K23
89^*f*^	—	KL110	KL110	KL110
93	K60	KL112	**KL112**	KL112^*d*^
94	—	KL62	KL62	K62
101	K24	KL24	KL24	K24
137	K17	KL17	KL17	K17^*d*^
143	—	KL151	KL151	KL151
150	—	KL163.KL27.KL46	—	—^*g*^
151	—	KL48	KL48	—
154	—	KL107	KL107	KL107
200^*f*^	—	—	—	—
201^*f*^	—	KL60	KL60	KL60
202^*f*^	—	KL127.KL155	KL127	KL127

a Known associated K types; serological reaction tested by Brisse et al. (14).

b KL, K locus; associated *cps* cluster type.

c The KL types confirmed by WGS results are in boldface font, the remaining are based in frequency distribution (KL127).

d Presence of outliers outside the main cluster (referred to as exception isolates in the manuscript).

e —, not defined.

f New *wzi* alleles submitted to BIGSdb.

g According to FT-IR results, we can discard the possibility of being KL27.

### (ii) Capsular prediction based on *wzy* or epidemiological data.

Some of these uncertain K types were additionally defined by sequencing of another molecular marker (*wzy*), by analysis of available epidemiological data (where the most frequently reported K type for a given sequence type [ST] was considered), and by WGS and Kaptive (see below) (Table 2) (7, 15, 16). Sequencing of *wzy* allowed distinguishing K14/K64 predicted by *wzi*64, whereas the epidemiological information (coupled with WGS in most of the cases) supported the prediction of capsular types K2, K16, K24, KL106, KL112, and KL127. With this approach, a total of 19 different K types were predicted by *wzi/wzy* sequencing that varied in frequency between 0.7% to 17.7% (Table 1). Twelve of them belong to serologically defined K types (K2, K14, K16, K17, K19, K23, K24, K27, KL48, KL60, KL62, and K64) and 7 are K types presumptively associated with a new composition/structure (KL105, KL106, KL107, KL110, KL112, KL127, and KL151) (Table 1).

It is of interest to highlight that most K types identified in this collection were specifically and uniquely associated with evolutionarily related strains from different countries and recovered from extended periods of time (Table 1). Some of them correspond to well-established clades from ST11/CG258 (7, 21, 28), CG15, CG14 (7, 20, 29), and ST258/CG258 (19, 30) identified in previous studies. Occasionally, the same K type was observed in different clones (e.g., K24 in ST11 and ST15, or K64 in ST11 and ST147) (Table 1).

### Molecular genotypic characterization of K. pneumoniae O antigen.

Considering that the O antigen can in some isolates protrude to the bacterial cell surface depending on the amount and type of the capsule, we cannot disregard its potential contribution to the biochemical makeup of the bacterial cell surface. In this sense, a molecular genotypic PCR-based approach was used to identify the most frequent O types previously recognized among K. pneumoniae clinical isolates (O1, O2, O3, O4, and O5) (10). As expected, the collection analyzed had much less O-type diversity. Most isolates belong to O1 (44.8% [69/154]) and O2 (39.6% [61/154]), followed by O4 (3.2% [5/154]), O3 (1.9% [3/154]), and O5 (0.7% [1/154]), while 9.7% (15/154) of the isolates were not typed with the primers used (Table 1). We also observed that isolates belonging to the same clone and exhibiting a given capsular type had the same O type, with very few exceptions. It is of note that evolutionarily related isolates belonging to the same or closely related clonal group, such as ST15 and ST14 from CG15/CG14 or ST11 and ST258 from CG258, exhibited the same O type (O1 for CG15/CG14 and O2 for CG258) (Table 1).

### Whole *cps*-based K-type assignments.

**FIG 1:**
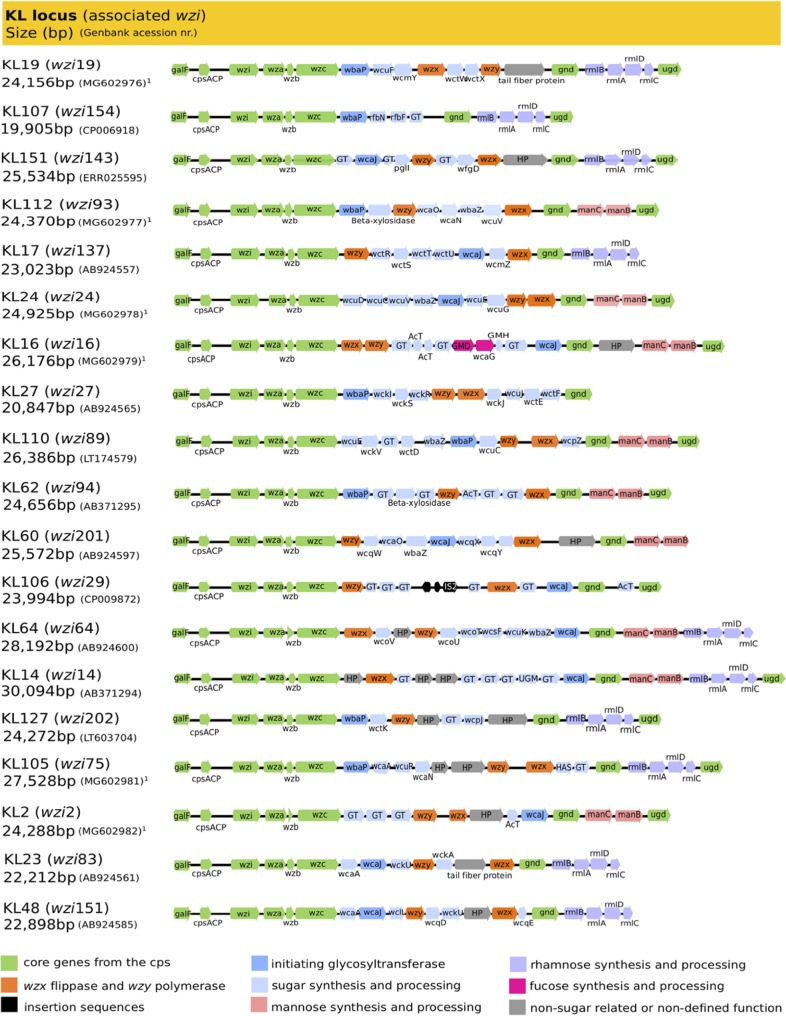
Representation of the *cps* loci identified in this study. Arrows indicate the direction, proportional length, and function (colored as per legend) of protein-coding genes. *cps* genetic clusters are labeled by the KL type; *wzi* alleles, size in base pairs, and GenBank accession numbers are indicated. ^1^, this study; HP, hypothetical protein; GT, glycosyltransferase; AcT, acyltransferase; GMD, GDP-mannose-4,6-dehydratase; GMH, GDP-mannose mannosyl hydrolase; HAS, hyaluronan synthase; UGM, UDP-galactopyranose mutase.

The whole *cps* cluster of the 19 *wzi*-defined K/KL types provided full resolution and supported the assignment of KL types for which the composition/structure is still unreported. Furthermore, it allowed us to detect changes in sites of the *cps* locus other than *wzi* or *wzy* that may influence the final capsule composition. We used *cps* of the reference K. pneumoniae collection available at the NCBI GenBank database and performed *de novo* whole-genome sequencing of 9 isolates from this study (Fig. 1). The isolates harboring *wzi*150 and *wzi*200 were excluded because they are underrepresented and the K/KL type was unclear or unknown (Table 2).

*cps* clusters represented in Fig. 1 presented a variable size (20 to 30 kb) and were delimited by the conserved *galF* (encodes a UTP-glucose-1-phosphate uridylyltransferase responsible for the synthesis of UDP-g-glucose) and *ugd* (encodes a UDP-glucose 6-dehydrogenase, responsible for the formation of UDP-d-glucoronate) genes. Each of the *cps* loci represented contains a unique combination of genes that is predictive of 19 different K/KL types. Whole *cps*-based typing allowed (i) confirmation of K-type predictions based on *wzi* sequencing and available epidemiological data (e.g., K2, K16, or K24), (ii) clarification of discrepancies between K type and KL types (e.g., KL112), and (iii) unveiling of small genetic differences in other sites of the locus that were subsequently correlated with biochemical changes detected by FT-IR (e.g., KL105 isolates [see below]). Thus, our data confirm that WGS provides a higher resolution for K. pneumoniae K typing but also that genomics data might be insufficient to precisely predict final capsule composition. It is also of remark that *cps* genes sequenced in this study were identical (nucleotide identity alignment, 100% to 99%) to those reported in isolates from the reference collection or previously deposited in public databases (see Table S1 in the supplemental material).

10.1128/mSystems.00386-19.4 TABLE S1 Reference capsular (K) types included in this study. Download Table S1, PDF file, 0.1 MB. Copyright © 2020 Rodrigues et al. 2020 Rodrigues et al. This content is distributed under the terms of the Creative Commons Attribution 4.0 International license.

### (i) Analysis of *cps* genes involved in sugar synthesis.

In a close analysis of all *cps* clusters, special attention was paid to the presence of genes associated with the synthesis of particular sugars: (i) initial glycosyltransferases responsible for triggering capsule synthesis. The *wbaP* (encoding an undecaprenyl phosphate galactose transferase) and *wcaJ* (encoding an undecaprenyl-phosphate glucose-1-phosphate) genes were detected in 8 or 9 of the *cps* clusters, respectively. The corresponding proteins revealed a high degree of homology (∼70% identity) and are, respectively, predictive of the presence of galactose or glucose on the repeat unit (11). (ii) Genes responsible for the synthesis of l-fucose (*gmd* and *wcaG*; *n* = 1/19 [5%]), GDP-d-mannose (*manCB*; *n* = 9/19 [47%]), and UDP-l-rhamnose (*rmlBADC*; *n* = 10/19 [53%]) were identified in the variable regions between *wzc* and *gnd* or between *gnd* and *ugd*. A series of other genes encoding putative noninitial glycosyltransferases, modifying enzymes (acetyltransferases, pyruvyl transferases, and glycosyl hydrolases), insertion sequences (IS), or hypothetical proteins were also detected (Fig. 1). These genotypic data supported correlations established with the presence of different sugars in the final capsule polysaccharide and predictions of the composition of unknown capsular types (see below).

### Differentiation of K types by FT-IR spectroscopy.

FT-IR spectroscopy detects variation of the vibrational modes of chemical bonds that are exposed to infrared radiation, and when applied to bacterial cells, it provides a highly specific whole-organism fingerprint that reflects their biochemical composition (22). The methodology we used is simple and inexpensive, since one bacterial colony is directly applied to an instrument with small amounts of consumables and low maintenance (see Materials and Methods for further details). Moreover, the time to result is very short, since one isolate can be typed in ca. 5 to 10 min at a lower cost (from 30%) than with competing DNA-based methods (22). Hence, we evaluated the ability of this methodology to differentiate the 19 K. pneumoniae K types or other surface structures. We compared spectra from all corresponding isolates obtained under the same experimental conditions and analyzed spectral variance by multivariate data analysis (see Materials and Methods for further details).

**FIG 2:**
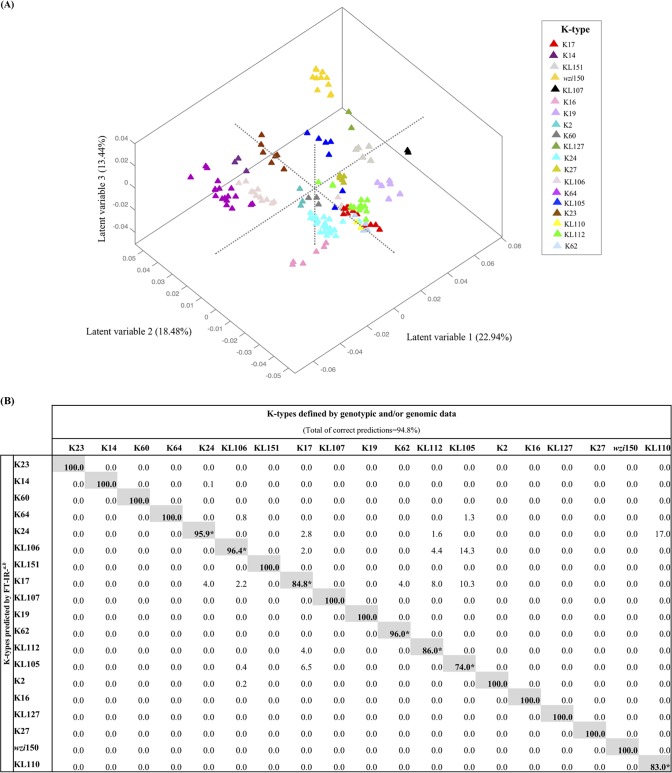
PLSDA model 1. (A) Score plot of the PLSDA regression model 1 according to K types corresponding to the first three latent variables (LVs). (B) Confusion matrix for K. pneumoniae PLSDA model 1 according to K types defined by genotypic and/or genomic data (values are percentages). *^a^*, gray shading represents the percentage of isolates correctly predicted using FT-IR for each K/KL-type; *^b^*, values obtained considering 20 LVs in PLSDA model (99.22% of variance covered); *, K types predicted by FT-IR with less than 100% of confidence are subsequently resolved in PLSDA model 2.

**FIG 3:**
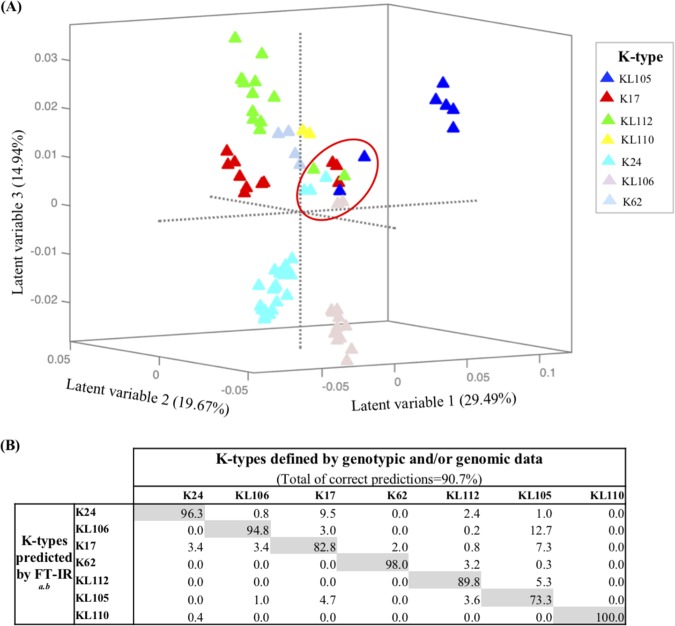
PLSDA model 2. (A) Score plot of the PLSDA regression model 2 according to K types corresponding to the first three latent variables (LVs). The red circle includes the isolates that have a different phenotypic behavior from their main class, referred to as exception isolates in the manuscript. (B) Confusion matrix for K. pneumoniae PLSDA model 2 according to K types defined by genotypic and/or genomic data (values are percentages). *^a^*, gray shading represents the percentage of isolates correctly predicted using FT-IR for each K type; *^b^*, values obtained considering 11 LVs (97.84% of variance covered) in PLSDA model.

**(i) General features of FT-IR spectral data.** FT-IR spectra of all K. pneumoniae isolates displayed typical bacterial bands that were previously related with the presence of different biomolecules such as lipids (W_1_, 3,000 to 2,800 cm^−1^), proteins/amides I and II (W_2_, 1,700 to 1,500 cm^−1^), phospholipids/DNA/RNA (W_3_, 1,500 to 1,200 cm^−1^), polysaccharides (W_4_, 1,200 to 900 cm^−1^), and a fingerprint region (W_5_, 900 to 700 cm^−1^) (31). The highest spectral variance was detected in the region dominated by vibrations of carbohydrates (W_4_, 1,200 to 900 cm^−1^) that was selected for spectral data analysis. Since the capsule is the most variable surface structure and it is mainly composed of polysaccharides, spectral diversity was analyzed and represented in supervised models considering 19 K types as classes. Two consecutive partial least-squares discriminant analysis (PLSDA) models were used to obtain the highest level of correct predictions for all classes (Fig. 2 and 3). In these models, we observed several well-defined clusters of isolates that were absolutely consistent with the K type. To further evidence that FT-IR spectroscopy differentiation is based on K-type variation, we developed an additional PLSDA model using STs as classes (see Fig. S1). In this model, we also observed several well-established clusters, some of them containing more than one ST if they shared the same K type (Fig. S1).

10.1128/mSystems.00386-19.1 FIG S1 Score plot of the PLSDA regression according to STs corresponding to the first two latent variables (LVs). Download FIG S1, TIF file, 0.6 MB. Copyright © 2020 Rodrigues et al. 2020 Rodrigues et al. This content is distributed under the terms of the Creative Commons Attribution 4.0 International license.

**(ii) Full K-type resolution in two PLSDA models.** In model 1 (19 classes modeled) (Fig. 2A), 12 clusters of isolates exhibiting 12 different K types were perfectly distinguished with 100% of total correct K-type predictions (Fig. 2B). These clusters included isolates belonging to O1 or O2 (e.g., K64 isolates). In fact, O1 and O2 have highly similar structures that are most probably indistinguishable by FT-IR spectroscopy. They are both composed of galactose homopolymers (alternating β-d-Galf and α-d-Galp residues) named d-galactan I (gal-I) or d-galactan III (gal-III) (O2 serogroup), which when capped with the gal-II (an antigenically different α-d-Galp and β-d-Galp disaccharide), form the O1 serogroup. Classes whose prediction rates were less than 100% (*n* = 7; K17, K24, K62, KL105, KL106, KL110, and KL112) were modeled independently using a second PLSDA (model 2). In model 2 (Fig. 3A), these 7 classes were well distinguished with an improved (0.4% to 17%) proportion of correct predictions (Fig. 3B). In fact, lower prediction rates were observed in heterogeneous classes that included *a priori* a few isolates that revealed a different biochemical profile than that expected for the respective class (designated “exception isolates”) (Fig. 3A; Table 2, and see below). A purge of these isolates increased the correct prediction rates toward 99.7% (see Fig. S2).

10.1128/mSystems.00386-19.2 FIG S2 PLSDA model 2 with no exception isolates. (A) Score plot of the PLSDA regression model 2 according to K types corresponding to the first three latent variables (LVs). (B) Confusion matrix for K. pneumoniae PLSDA model 2 according to K types defined by genotypic and/or genomic data (values are percentages). *^a^*, gray shading represents the percentage of isolates correctly predicted using FT-IR for each K type; *^b^*, values obtained considering 10 LVs in PLSDA model. Download FIG S2, TIF file, 0.6 MB. Copyright © 2020 Rodrigues et al. 2020 Rodrigues et al. This content is distributed under the terms of the Creative Commons Attribution 4.0 International license.

Thus, the FT-IR-based typing method discriminated the 19 different K types tested, supporting differences in their final capsule composition or structure, including the biochemically uncharacterized KL types. It provided a resolution identical to that of whole *cps* sequencing for discriminating closely related K types (K14 and K64) or discrepant K/KL types (KL60 and KL112) (Table 2). Moreover, not only were precise biochemical-genotypic correlations established, but also, this methodology depicted differences in a few exception isolates (8.9% [12/152]) that were not predicted by molecular genotypic data. These isolates presented changes in sites of the locus that were not detected or could be neglected by genotypic approaches (Tables 1 and 2; see also below).

**FIG 4:**
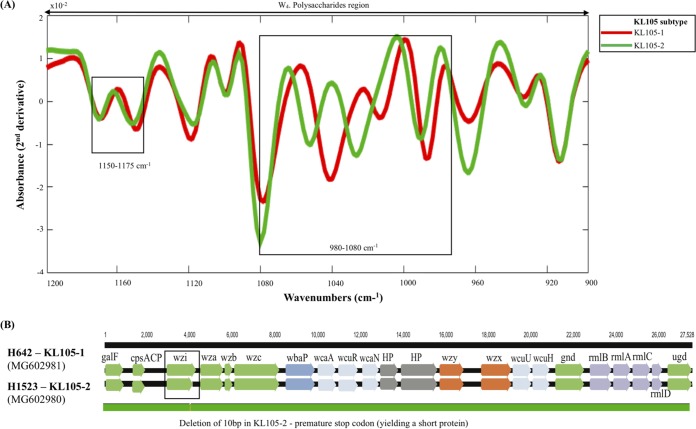
Spectral and genetic representation of KL105 K. pneumoniae subtypes. (A) K. pneumoniae KL105 FT-IR mean spectra in the region 900 to 1,200 cm^−1^. The most discriminatory spectral regions between the two KL105 subtypes are delimited by black squares. (B) Representation of *cps* loci of KL105-1 and KL105-2 isolates. Arrows indicate the direction and proportional length of protein-coding genes. *cps* genetic clusters are labeled by the KL subtype and GenBank accession numbers are indicated. HP, hypothetical protein.

**(iii) Exploring capsular discrepancies between genotypic methods and FT-IR.** First, one KL112 isolate (ST17) incorrectly predicted might represent one of the few cases where differences in the O type might impact on FT-IR spectra. This isolate was classified as O5, which is composed of a homopolymer of mannose (instead of d-galactan from O1/O2) yielding a different polysaccharide configuration (17, 32). Second, 2 KL105 ST11 isolates (arbitrarily designated KL105-2) were distinguished by FT-IR spectroscopy from the others predicted as KL105 (arbitrarily designated KL105-1) (Fig. 3A), though all of them share identical pulsed-field gel electrophoresis (PFGE) types and are epidemiologically related (Table 1) (33). Differences in the spectra were observed in the 1,080 to 980 cm^−1^ region, supported by a 10-bp deletion within the *wzi* in KL105-2 that was detected by comparison of the whole *cps* operon (27 kb in size) (Fig. 4). This deletion is outside the region sequenced by *wzi*-based typing and results in a Wzi protein with 427 amino acids (aa; instead of 477 aa), probably affecting final capsule composition or amount (Fig. 4B) (34). Third, one K24 isolate (H1119) predicted by *wzi* sequencing had a recombinant K24/K39 *cps* locus (sequence was deposited in GenBank database with accession number NXBL00000000). Fourth, FT-IR detected differences in 2 KL106 isolates. An *in silico* analysis of 496 ST258 genomes (publicly available at NCBI in February 2018) by Kaptive revealed that 153 of them (31%) carried *wzi*29 and were predicted as KL106. In a deeper analysis, we depicted two main KL106 subtypes circulating (arbitrarily named KL106-1 [92/153] and KL106-2 [50/153]) that differed in the presence or absence of an IS*5* sequence (KL106-2 + 1,200 bp) upstream of the *wzi* gene (Fig. S3). Thus, we hypothesize that the two clusters depicted by FT-IR for KL106 might represent these two variants.

10.1128/mSystems.00386-19.3 FIG S3 Genetic representation of the two KL106 subtypes, KL106-1 and KL106-2, identified among publicly available ST258-*wzi*29 K. pneumoniae genomes. Arrows indicate the direction and proportional length of protein coding genes. *cps* genetic clusters are labelled by the strain name and KL subtype. GenBank accession numbers are indicated. GT, glycosyltransferase; AcT, acetyltransferase. Download FIG S3, TIF file, 0.8 MB. Copyright © 2020 Rodrigues et al. 2020 Rodrigues et al. This content is distributed under the terms of the Creative Commons Attribution 4.0 International license.

Thus, FT-IR spectroscopy can reliably detect differences in capsule composition of main K. pneumoniae K types, providing a resolution identical to or even greater than that of one of the most discriminatory genotypic-based K-typing methods (WGS).

### Statistical analysis.

The discriminatory power of FT-IR was calculated by using the Simpson’s index of diversity (SID) applied to the test population for all the typing methods considered (FT-IR, MLST, *wzi* sequencing, and epidemiological data). The SID for FT-IR was 0.932, a higher value than those obtained for *wzi* sequencing (0.918) or epidemiological data (0.916) (see Table S2). To assess the congruence between the typing methods, we calculated the Wallace coefficient (see Table S3). This coefficient reflects the likelihood of two isolates assigned to the same type by one method (e.g., FT-IR) being classified together using another typing method (e.g., *wzi* sequencing). The high coefficients for FT-IR and epidemiological data (1.000) and for FT-IR and *wzi* (0.966) indicate that the combination of either epidemiological data or *wzi*-based K-type predictions to FT-IR adds no or little additional strain discrimination. Furthermore, the chance that two isolates sharing the same FT-IR type also shared the same ST is lower (67.5%), reflecting the lower discriminatory power of MLST.

10.1128/mSystems.00386-19.5 TABLE S2 Simpson’s index of diversity of the different typing methods. Download Table S2, PDF file, 0.1 MB. Copyright © 2020 Rodrigues et al. 2020 Rodrigues et al. This content is distributed under the terms of the Creative Commons Attribution 4.0 International license.

10.1128/mSystems.00386-19.6 TABLE S3 Congruence between typing methods determined by adjusted Wallace coefficient. Download Table S3, PDF file, 0.1 MB. Copyright © 2020 Rodrigues et al. 2020 Rodrigues et al. This content is distributed under the terms of the Creative Commons Attribution 4.0 International license.

### Correlation between FT-IR K types and capsule biochemical composition.

**FIG 5:**
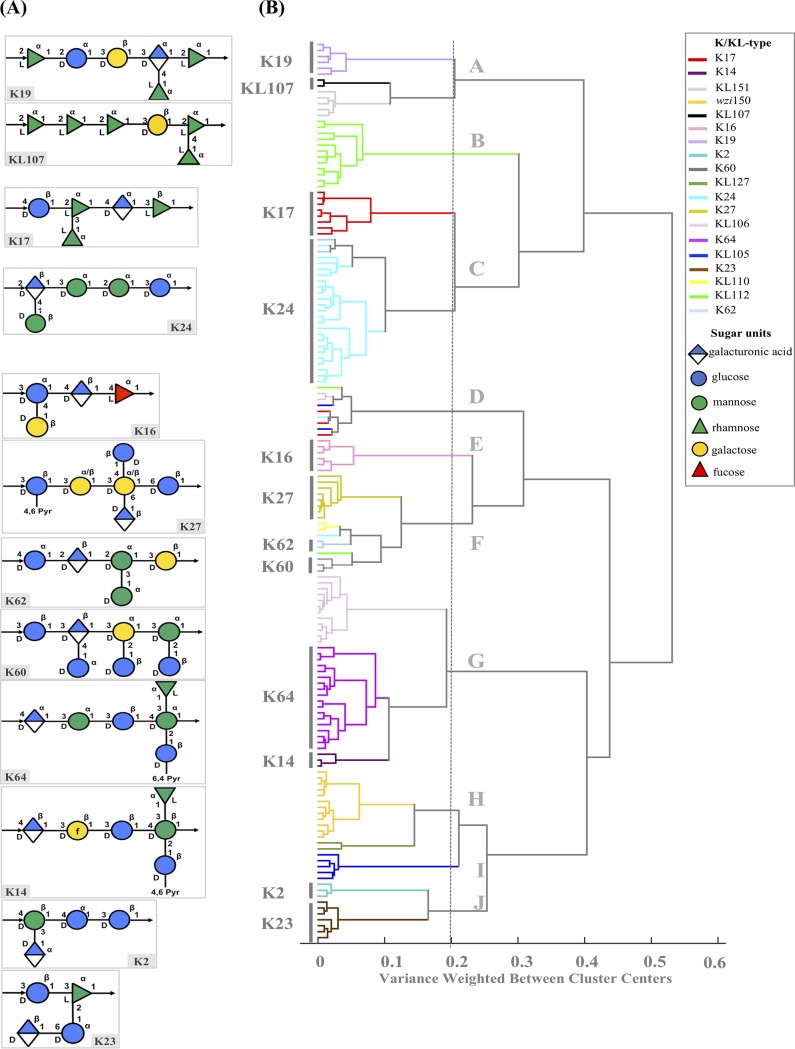
Clustering and biochemical composition and of K/KL types detected in this study. (A) Known CPS biochemical structures of K. pneumoniae included in this study. The structures were represented using https://iith.ac.in/K-PAM/k27.html and in accordance with the symbol nomenclature for glycans (SNFG) (https://www.ncbi.nlm.nih.gov/glycans/snfg.html). Arrows represent the type of linkages between the monosaccharides within the K unit. f, furanose ring. (B) Dendrogram obtained from the 1,200 to 900 cm^−1^ spectral region using Ward’s algorithm and 13 principal-component (PC) distance for isolates of the different K/KL types.

To unequivocally settle the basis for FT-IR-based K-type discrimination, we represented the similarity of the spectra in a dendrogram generated by hierarchical cluster analysis (HCA) and correlated the FT-IR-based assignments with the biochemical composition of the different known K types (Fig. 5). In this figure, we can see that the same 19 K types were also discriminated in clusters defined at distances of <0.4 (Fig. 5A). In parallel, we represented in Fig. 5B the composition and structure of 12 of 19 known K types, for which their source information is included in Table S1. We observed that these capsular types exhibit a marked diversity of patterns based on the size of the polysaccharide polymer, the number and type of monosaccharides, the type of linkages or the presence of side chains, or modifications of the lateral sugars that are on the basis of correct FT-IR-based discrimination. They vary between tetra- and heptasaccharides made up of glucose, glucuronic acid, mannose, rhamnose, fucose, galactofuranose, or galacturonic acid in different proportions and orders, though some appear to have similar structures (Fig. 5B).

**(i) Analysis of similar K types inferred from FT-IR spectra.** A high similarity between K types K19 and KL107 (Fig. 5A, branch A), K17 and K24 (Fig. 5A, branch C), K14 and K64 (Fig. 5A, branch G), and K2 and K23 (Fig. 5A, branch J) is inferred from the HCA (distances of <0.2), which is supported by their closely related K-type structures, as explained below (Fig. 5B).

**(ii) K19 and KL107.** These capsular types are both composed of hexasaccharides that have in common a high number of rhamnose residues (3 and 5, respectively) and vary slightly in the compositions of other sugars. Whereas K19 contains a polymer of d-galactose, d-glucose, l-rhamnose (3 monomers), and d-glucuronic acid, KL107 contains d-galactose, d-galacturonic acid, and rhamnose (4 monomers).

**(iii) K17 and K24.** These capsular types consist of similar pentasaccharide structures, containing d-glucose, d-glucuronic acid, and 3 l-rhamnose (K17) or 3 d-mannose (K24), that differ only in the final hydroxyl group.

**(iv) K14 and K64.** They are composed of highly similar hexasaccharides composed of d-glucose (2 monomers, one of them acetylated), d-glucuronic acid, l-rhamnose, and either 2 d-mannoses (K64) or 1 l-mannose and 1 galactofuranose (K14). In fact, both structures are highly similar even in configuration, and these K types yield cross-reactions in serological methods.

**(v) K2 and K23.** These two capsular types are characterized by tetrasaccharides in different configurations, composed of d-glucose (2 monomers) and d-glucuronic acid and either d-mannose (K2) or l-rhamnose (K23) that are highly similar sugars differing only in the conformation and the final group (CH_3_ or CH_2_OH).

Additionally, KL60, KL62, and K27 were all grouped in branch F from Fig. 5A, which also included a few K types for which the structure is not known, and for this reason, any comparison lacks robustness. We observed that KL60, KL62, and K27 are diverse in structure (penta-heptasaccharides) and composition (variable but especially enriched in glucose). K16 appears in a separate branch (E) from the dendrogram and is clearly distinguished from all the others, since it is formed by a tetrasaccharide containing d-glucose, d-glucuronic acid, and d-galactose, and it is the only one containing l-fucose.

The correlations established strengthen FT-IR-based K-type assignments and highlight the need to both characterize the structure/composition of new KL types and increase the reliabilities of the clustering and the comparisons with a higher number of isolates from certain K types.

### Prediction of the capsular composition based on FT-IR spectroscopy assignments.

Several K types included in this study are observed in worldwide-spread K. pneumoniae lineages (KL105, KL106, KL110, KL112, KL127, and KL151) encountered among MDR K. pneumoniae clinical isolates for which the structure has not yet been characterized. In this section, we provide insights into the possible structure and composition (type of sugars) for these new KL types considering the similarity between FT-IR spectra (Fig. 5A) combined with the information contained in their corresponding *cps* loci (Fig. 1).

Since spectra obtained from isolates exhibiting KL105 and KL127 clustered with K2 and K23 types (distance < 0.3), we predict a tetrasaccharide structure composed of d-glucose, d-glucuronic acid, and possibly d-galactose and d-rhamnose, which is also supported by the presence of genes *wbaP* and *rmlBADC*, respectively, in the *cps* operon. The KL112 capsule is closely related to that of K17 or K24 at a distance of >0.3. Thus, we predict that it could be composed of a pentasaccharide of d-glucose, d-galactose, and d-mannose, which is also corroborated by the presence of *wbaP* and *manCB* on the *cps* cluster. Similarly, the KL151 capsular type is highly related with KL107; thus, we expect it to be a hexapolysaccharide composed of several rhamnose residues (supported by the presence of *rmlBADC*) and the absence of d-galactose (*wcaJ* instead of *wbaP*). KL110 might be a pentasaccharide comprising several units of d-glucose and/or d-galactose and mannose according to the corresponding operons encountered in the *cps* operon.

Thus, using our FT-IR-based framework, we predicted for the first time the presumptive structure/composition of new KL types, which was supported by *cps* genotypic data. Further studies are needed to validate these predictions and potentiate the use of FT-IR spectroscopy for K-type identification and characterization.

## DISCUSSION

In this study, we establish for the first time a framework to support FT-IR spectroscopy as an accurate, simple, quick, and inexpensive method for the characterization and identification of K. pneumoniae capsular types. The multidisciplinary strategy used allowed clarification of the fundamentals for FT-IR-based K-type discrimination, increased knowledge of K-type variation (especially on the newly described KL types), and validation of the methodology for K typing, which can be extremely useful for outbreak management and epidemiological surveillance of MDR K. pneumoniae. We demonstrated that FT-IR-based K-type differentiation relies on the distinctive biochemical profiles obtained in the spectral region dominated by carbohydrates (W_4_, 1,200 to 900 cm^−1^) (31). It was further substantiated with whole *cps* locus analysis and with the number and type of monomers that make up the capsular polysaccharide, whereas the order and type of bonds (alpha or beta) does not seem to influence K-type prediction. The same spectral region was previously reported to be highly discriminatory for several bacterial species, including other relevant clinical or food pathogens such as Escherichia coli, Acinetobacter baumannii, Salmonella enterica, Staphylococcus aureus, or Streptococcus pneumoniae (24–26, 35, 36). Some of these studies have also pointed out a correlation with variation on bacterial serogroups or capsules, supporting the potential to extend the approach developed here to other bacterial pathogens of interest to clinical or food microbiology (22).

In fact, the importance of surface structures (and especially the capsule) on evolution, pathogenesis, and host adaptation of bacterial pathogens is well known. However, full understanding on K-type variation in K. pneumoniae has been hindered by variable drawbacks of available methods for K typing and reawakened only with the burst of genotypic or genomics-based approaches (17, 37). In fact, the putative number of capsule types encountered varies strongly with the method used. Until now, there have been 655 *wzi* and 121 *wzc* alleles recognized (http://bigsdb.pasteur.fr/klebsiella/klebsiella.html), the latter probably less affected by recombination events. On the other hand, 161 *cps* locus (KL) types have been predicted by whole *cps* sequencing, which is twice the number of K types initially recognized by traditional serotyping techniques (77 K types) (7). In the absence of structural data on these new K types, it remains to be clarified if all of them correspond to biochemically distinct types or their correlation with the 77 K types of reference. Our FT-IR-based approach supports a unique capsular structure for each one of the K/KL types tested, including closely related ones (e.g., K14 and K64). In this sense, FT-IR spectroscopy could be extremely useful in the validation of the new inferred KL types and guide the selection of those to be characterized biochemically, a highly desired goal (37). In addition, it unveiled biochemical differences within isolates considered from the same K type by genotypic methods (e.g., KL105 or KL106), whose significance is unclear when only genomic data are considered, reinforcing the sensitivity of the method. The frequency with which these phenomena occur in the clinical setting, the underlying reasons for, or even their significance in the context of host interactions are unknown, but they seem to occur sporadically (38–40). In some cases, they might represent important evolutionary steps with several biological consequences, as occurred with the two different ST258 clades exhibiting KL106 or KL107 K types (39, 40).

Currently, the correct prediction of K types in K. pneumoniae depends on a combined approach of genetic markers, epidemiological data, and/or comparative genomics of the *cps* locus, which is not straightforward and requires expertise. Furthermore, the gap between genetically or serologically defined K types is still a problem. We propose here the use of FT-IR spectroscopy to bridge this gap and support biochemical characterization of K. pneumoniae K types. Besides, it gathers essential features for a reliable, easy-to-use, fast, and low-cost solution for routine K typing.

The ability of FT-IR spectroscopy to discriminate and identify K. pneumoniae capsular types represents a major advantage, since K types were found to be good epidemiological markers of particular K. pneumoniae lineages with clinical and biological significance. Detailed and comprehensive phylogenomics studies have shown a high specificity between certain lineages and particular K types within CG14, CG15, or CG258 causing hospital- or community-acquired infections worldwide (7, 14, 15, 19, 20, 28). It was based on this information that we selected representative widespread MDR lineages characterized at the genomics and/or molecular level to include in this study and showed that they were correctly depicted by FT-IR spectroscopy, reflecting the high discriminatory power of the methodology.

Our in-house FT-IR K. pneumoniae models are being used routinely and successfully to classify unknown K. pneumoniae isolates, where >500 isolates from different hospitals, long-term-care facilities, and community laboratories have already been tested. The spectra obtained from new isolates are compared with our own databases, and their projection on the PLSDA models generated in this study provides a tentative K-type assignment, which is being corroborated by *wzi* sequencing and/or WGS (data not shown). On this basis, the method has been crucial in the recognition and early detection of several hospital outbreaks involving carbapenemase (KPC-3, OXA-48, and NDM-1) and extended-spectrum β-lactamase (ESBL) and/or MCR-1 producers, where useful information (K type and tentative clone assignment according to local epidemiology) is sent in a short time (from 1 h depending upon the number of isolates) provided that bacteria are grown under standardized conditions (41, 42). Under our experimental conditions, using a total attenuated reflectance (ATR) FT-IR and bacteria directly on the target, K-typing information is obtained at 35% of the cost of *wzi* sequencing. This proportion is even lower when we compare with gold standard reference methods for strain typing such as PFGE (3%) or WGS (1%), which is highly attractive for routine applications. Reproducible results were obtained with the same instrument as well as with FT-IR equipment from different manufacturers (Frontier or Spectrum 2 from Perkin-Elmer and FT-IR Alpha from Bruker) and also using variable experimental conditions (±4 h of incubation time, different culture media) (22), ensuring the stability of the method under variable environmental conditions.

IR spectra can also be used for bacterial differentiation at the species level, but there are not yet reliable databases (23). Thus, in clinical microbiology laboratory routines, we envision that FT-IR spectroscopy can be used downstream of matrix-assisted laser desorption ionization–time of flight mass spectrometry (MALDI-TOF MS) species identification for quick detection of K. pneumoniae outbreak strains or monitoring epidemiological trends, for simple and quick capsular typing, or as a screening tool to select representative isolates for whole-genome sequencing. Compared to that for other spectroscopic methods, FT-IR spectroscopy has higher resolution than MALDI-TOF MS for strain typing (including for species of clinical interest) and a much higher sensitivity and reproducibility than Raman (22).

We recognize that the coverage of our database needs to be enlarged to represent as much K-type diversity as possible toward a clinical application in a wider epidemiological context. Also, strain typing will always depend on the stability of strain capsular traits and the establishment of reliable genotypic-biochemical correlations. For routine clinical applications, the method needs to be adapted for a nonspecialist user, which depends on the creation of judicious databases under standardized conditions and automation of data analysis. This problem was partially solved by Bruker, who launched in June 2016 a dedicated FT-IR-based equipment (IR Biotyper) for routine outbreak detection using a simple and automated process.

## MATERIALS AND METHODS

### Bacterial strains.

One-hundred fifty-four well-characterized MDR K. pneumoniae clinical isolates representing main clonal groups (CG) circulating in different geographic regions (Brazil, Greece, Poland, Portugal, Romania, and Spain) for long periods of time (2002 to 2015) were selected to validate the approach. Clonal relatedness among the isolates was evaluated by gold standard and reference genotypic methods (multilocus sequence typing and pulsed-field gel electrophoresis), depicting 13 STs (representing 7 CG) and 34 PFGE types. Most of the isolates were producers of ESBLs, acquired AmpCs and/or carbapenemases, and were enriched in particular virulence factors, such as the urease cluster (100%), type 1 and 3 fimbriae (99.4%), and the yersiniabactin siderophore (*ybtS*) and iron transporter permease genes (*kfuBC*) (64% and 37%, respectively), the latter with variable distribution in the collection analyzed. Details about the bacterial isolates included in this study are summarized in Table 1 as well as Table S4 in the supplemental material.

10.1128/mSystems.00386-19.7 TABLE S4 Characteristics of the 154 international MDR K. pneumoniae clinical isolates analyzed in this study. Download Table S4, PDF file, 0.2 MB. Copyright © 2020 Rodrigues et al. 2020 Rodrigues et al. This content is distributed under the terms of the Creative Commons Attribution 4.0 International license.

### Genotypic and phenotypic characterization of surface polysaccharide structures.

In all isolates, PCR and sequencing of specific genetic markers were used for genotyping of K and O types. For the genotypic-based prediction of K types, we sequenced a 447-bp fragment from a highly variable region of *wzi* and, occasionally, specific *wzy* fragments (14, 15). Regarding O genotyping, specific regions of *wzm* and *wzt* genes from the *rfb* cluster were amplified for O1/O2, O3, and O5 identification. Furthermore, an additional PCR was performed to distinguish O1 and O2 and its variants (designed in the *wbbY* loci unlinked to the *rfb* cluster) (10, 17). Additionally, WGS was performed for 9 isolates for which discrepancies between genotypic and biochemical data were observed. WGS was performed by Illumina MiSeq (2× 300-bp pair-ended runs, ∼6 Gb per genome, coverage 100×), and reads were assembled using SPAdes version 3.9.0 (cab.spbu.ru/software/spades/); the full *cps* locus was further annotated with Geneious R10 software (Biomatters Ltd., Auckland, New Zealand) considering the nomenclature proposed by Reeves et al. (43).

Biochemical characterization of surface bacterial components was performed using FT-IR spectroscopy with attenuated total reflectance (ATR) mode, as previously described (35, 36). Briefly, isolates were grown on Mueller-Hinton agar at 37°C for 18 h, and colonies were directly transferred from the agar plates to the ATR crystal and air-dried in a thin film. Spectra were acquired using a Perkin Elmer Spectrum BX FT-IR system spectrophotometer in the ATR mode with a PIKE Technologies Gladi ATR accessory from 4,000 to 600 cm^−1^, a resolution of 4 cm^−1^, and 32 scan coadditions. For each isolate, at least three instrumental replicates (obtained from the same agar plate in the same day) and three biological replicates (obtained in three independent days) were acquired and analyzed, corresponding to a minimum of nine spectra per strain (36, 44).

### Spectral data analysis.

All chemometric analyses was performed using Matlab R2015a version 8.5 (MathWorks, Natick, MA) and PLS Toolbox version 8.5 for Matlab (Eigenvector Research, Manson, WA, USA). Original FT-IR spectra were processed with standard normal variate (SNV) followed by the application of a Savitzky-Golay filter (9 smoothing points, second-order polynomial, and second derivative) (45, 46). Prior to modeling with PLSDA, spectra were mean centered. Due to the amount of generated data and for simplification of the visualization, a mean spectrum of each isolate (resulting from at least nine congruent replicates validated by a principal-component analysis [PCA]-based internal script). Spectra were analyzed by a supervised (partial least-squares discriminant analysis [PLSDA]) chemometric model using, for discriminatory purposes, the region of the spectra corresponding to the carbohydrate vibrations (W_4_, 1,200 to 900 cm^−1^) (31). PLSDA is a supervised method based on the PLS regression method. In PLSDA models, we assign to each isolate spectrum (*x_i_*) a vector of zeros with the value of 1 at the position corresponding to its class (*y_i_*, ST or K type) in such a way that categorical variable values (*y_i_*) can be predicted for samples of unknown origin. Model loadings and the corresponding scores were obtained by sequentially extracting the components or latent variables (LVs) from matrices *X* (spectrum) and *Y* (matrix codifying K types). In PLSDA, a probability value for each assignment is estimated for each sample. The number of latent variables (LVs) was optimized using the leave-one-sample-out cross-validation procedure in order to prevent overfitting, considering only 70% of the available data (randomly selected). After optimization of the number of LVs, the model was tested on the remaining 30% samples in order to assess the proportion (%) of correct predictions for each class (36, 44, 47). We used a 1,000× bootstrap for this procedure to ensure the robustness of this internal validation.

The unsupervised method hierarchical cluster analysis (HCA) was also applied to evaluate the spectral similarity between isolates (and eventually to correlate clusters with K-type structures). The dendrograms were obtained using Ward’s algorithm, as previously described (36). Thirteen components were retained with a total accumulated variance of 96.32%. The same preprocessing and scaling used for PLSDA was used for HCA.

The discriminatory ability of FT-IR spectroscopy compared to that of MLST, PFGE, *wzi* sequencing, and epidemiological data was measured using the Simpson’s index of diversity (SID) (Table S2). The congruence between the typing methods was calculated using the adjusted Wallace coefficient (Table S3). All calculations were conducted using the Comparing Partitions website (http://www.comparingpartitions.info/index.php?link=Tool). Pairwise comparisons were performed on data sets in which missing data (e.g., a K type could not be determined by one of the methods) were not considered.

### Data availability.

The sequences of the complete *cps* operon were deposited in the GenBank database under the accession numbers MG602975 to MG602982 and under the BioProject PRJNA408270. The sequence for K24 isolate (H1119) predicted by *wzi* sequencing with a recombinant K24/K39 *cps* locus was deposited in the GenBank database under accession number NXBK00000000).
