# Supplementary material for: A Front Line on Klebsiella pneumoniae Capsular Polysaccharide Knowledge: Fourier Transform Infrared Spectroscopy as an Accurate and Fast Typing Tool
Source: mSystems. 2020 Mar 24;5(2):e00386-19. doi: 10.1128/mSystems.00386-19 (PMC7093823; doi:10.1128/mSystems.00386-19)
Supplement: TABLE S1 [file mSystems.00386-19-st001.pdf]

Table S1. Reference capsular (K) types included in this study.

Capsular type	Strain	GenBank Accession no.	Reference
**K2**	VGH525	AB371296	[1]
**K14**	VGH916	AB371294	[2]
**K16**	2069/49	AB742228	[3]
**K17**	2005/49	AB924557	[4]
**K19**	293/50	AB924559	[5]
**K23**	2812/50	AB924561	[6]
**K24**	1680/49	AB924562	[7]
**K27**	6613	AB924565	[8]
**K60**	4463/52	AB924597	[9]
**K62**	VGH698	AB371295	[10]
**K64**	NCTC 8172	AB924600	[11]
**KL107**	2796	GCA_000281375.1	[12]

1. Gahan, L. C., Sandford, P. A. & Conrad, H. E. The Structure of the Serotype 2 Capsular Polysaccharide of Aerobacter aerogenes. *Biochemistry* **6**, 2755–2767 (1967).
2. Dutton, G. G. S., Parolis, H. & Parolis, L. A. S. A structural investigation of the capsular polysaccharide of *Klebsiella* K14. *Carbohydr. Res.* **140**, 263–275 (1985).
3. Chakraborty, A. K., Friebolin, H., Niemann, H. & Stirm, S. Primary structure of the *Klebsiella* serotype 16 capsular polysaccharide. *Carbohydr. Res.* **59**, 525–530 (1977).

4. Dutton, G. G. S. & Folkman, T. E. Structural investigation of the capsular polysaccharide of *Klebsiella* serotype K17. *Carbohydr. Res.* **52**, 147–161 (1995).

5. Beurret, M., Vignon, M. & Jean-Paul, J. Structural investigation of the capsular polysaccharide from *Klebsiella* K19 by chemical and N.M.R. analyses. *Carbohydr. Res.* **157**, 13–25 (1986).

6. Dutton, G. G. S., Mackie, K. L., Savage, A. V & Stephenson, M. D. Structural investigation of the capsular polysaccharide of *Klebsiella* serotype K23. *Carbohydr. Res.* **66**, 125–131 (1978).

7. Yuen-Min, C., Dutton, G. G. S. & Zanlungo, A. M. The Structure of the Capsular Polysaccharide of *Klebsiella* K-type 24. *Carbohydr. Res.* **73**, 169–174 (1979).

8. Churms, S. C., Merrifield, E. H. & Stephen, A. M. The molecular structure of the capsular polysaccharide from *KIebsielIa* type 27. *Carbohydr. Res.* **81**, 49–58 (1980).

9. Dutton, G. G. S. & Fabio, J. Di. The capsular polyssacharide of *Klebsiella* serotype K60; a novel structure pattern. *Carbohydr. Res.* **87**, 129–139 (1980).

10. Dutton, G. G. S. & Yang, M.-T. Structural investigation of *Klebsiella* serotype K62 polysaccharide. *Carbohydr. Res.* **59**, 179–192 (1977).

11. Merrifield, E. H. & Stephen, A. M. Structural studies on the capsular polysaccharide from *KIebsielIa* serotype K64. *Carbohydr. Res.* **74**, 241–257 (1979).

12. Kubler-Kielb, J. *et al.* The capsular polysaccharide and lipopolysaccharide structures of two carbapenem resistant *Klebsiella pneumoniae* outbreak isolates. *Carbohydr. Res.* **369**, 6–9 (2013).
