# Supplementary figures and images for: A Front Line on Klebsiella pneumoniae Capsular Polysaccharide Knowledge: Fourier Transform Infrared Spectroscopy as an Accurate and Fast Typing Tool
Source: mSystems. 2020 Mar 24;5(2):e00386-19. doi: 10.1128/mSystems.00386-19 (PMC7093823; doi:10.1128/mSystems.00386-19)

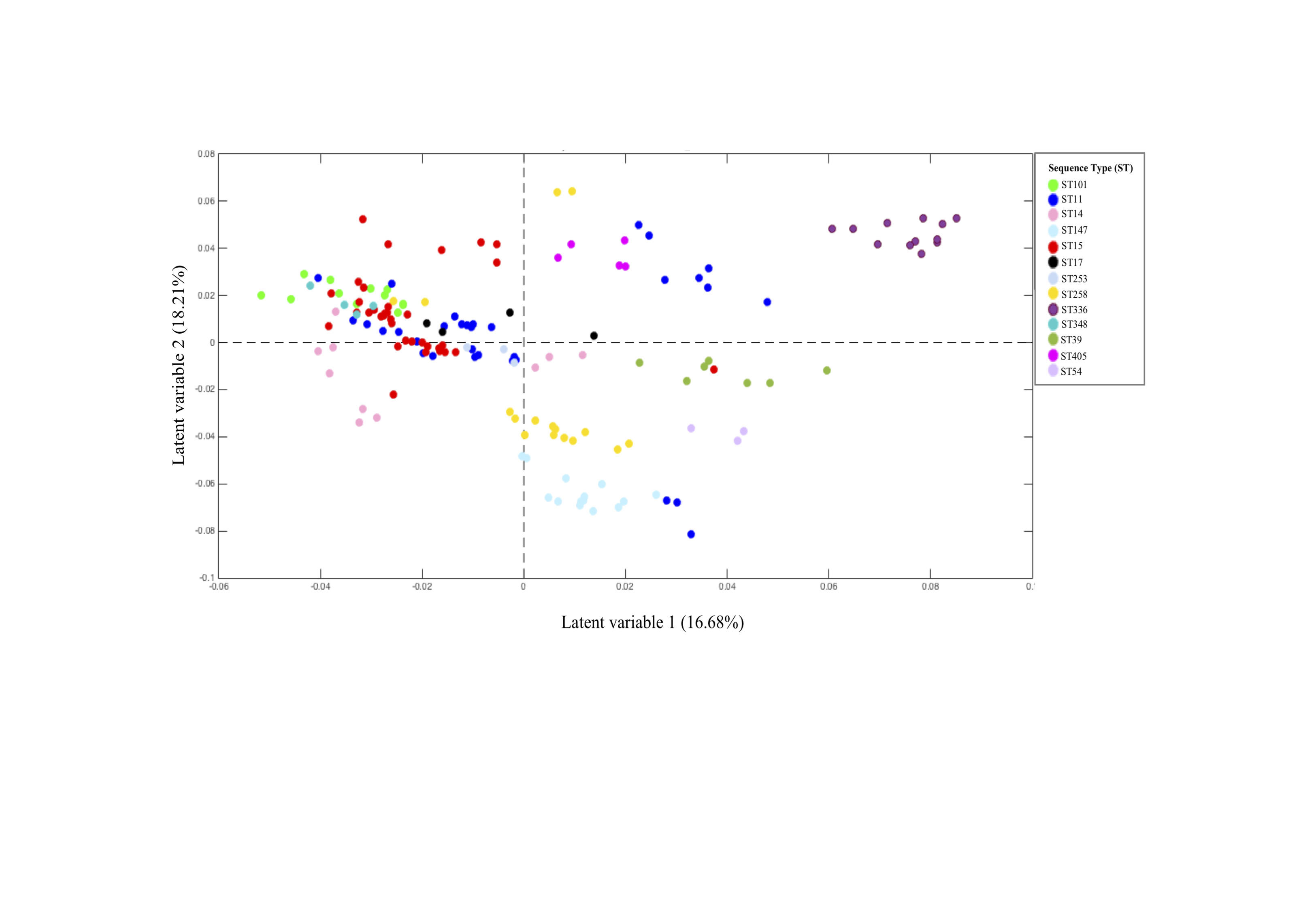

Supplement: FIG S1 [file mSystems.00386-19-sf001.tif]

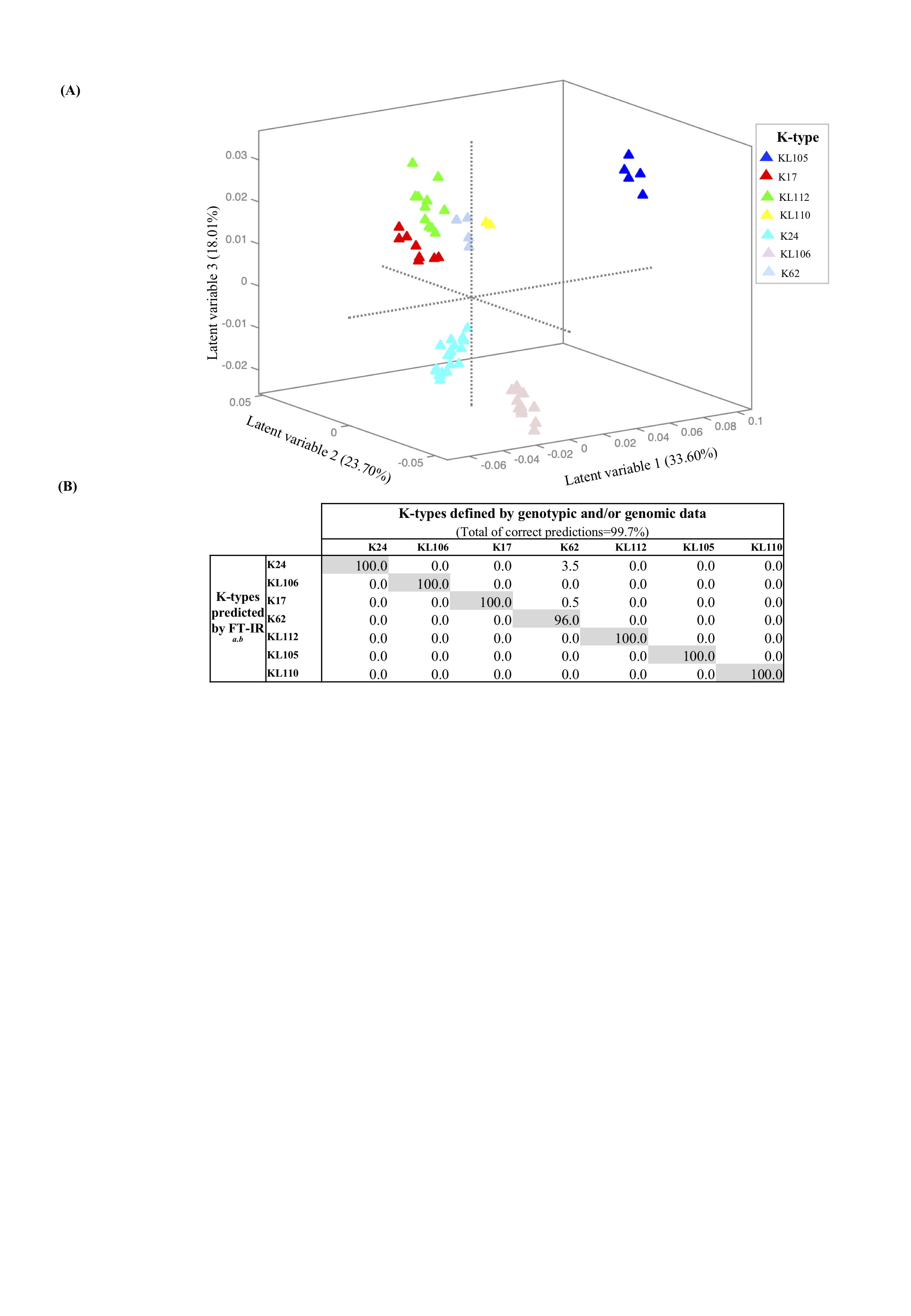

Supplement: FIG S2 [file mSystems.00386-19-sf002.tif]
