# Supplementary material for: A Front Line on Klebsiella pneumoniae Capsular Polysaccharide Knowledge: Fourier Transform Infrared Spectroscopy as an Accurate and Fast Typing Tool
Source: mSystems. 2020 Mar 24;5(2):e00386-19. doi: 10.1128/mSystems.00386-19 (PMC7093823; doi:10.1128/mSystems.00386-19)
Supplement: TABLE S2 [file mSystems.00386-19-st002.pdf]

Table S2. Simpson's Index of Diversity of the different typing methods.

Typing Method	SID[1] [95% CI]	No. of types[2]
FT-IR	0.932 [0.915-0.948]	26
Epidemiological Data[3]	0.916 [0.898-0.934]	18
wzi sequencing	0.918 [0.901-0.936]	19
MLST	0.861 [0.834-0.888]	12
PFGE	0.955 [0.945-0.965]	32

[1]Simpson's Index of Diversity, 95% CI, 95% confidence interval

[2] Only complete datasets for the four typing methods were considered – ST336-*wzi*150, ST15-*wzi*151 and ST17-*wzi*200 were not taken into account for this calculation.

[3]Hereby representing the frequency with which a given K-/KL-type has been described in the literature. In our collection, in case of multiple K-assignments, we considered the most frequent one and, in most cases, we confirmed it by WGS.
