# Supplementary material for: A Front Line on Klebsiella pneumoniae Capsular Polysaccharide Knowledge: Fourier Transform Infrared Spectroscopy as an Accurate and Fast Typing Tool
Source: mSystems. 2020 Mar 24;5(2):e00386-19. doi: 10.1128/mSystems.00386-19 (PMC7093823; doi:10.1128/mSystems.00386-19)
Supplement: TABLE S3 [file mSystems.00386-19-st003.pdf]

Table S3. Congruence between typing methods determined by adjusted Wallace coefficient.

	Adjusted Wallace coefficient [95% CI]				
**Typing Method**	**MLST**	***wzi* sequencing**	**Ep. Data**[1]	**FT-IR**	**PFGE**
**MLST**		0.371 [0.318-0.424]	0.379 [0.326-0.432]	0.307 [0.251-0.363]	0.290 [0.240-0.340]
***wzi* sequencing**	0.675 [0.607-0.743]		1.000 [1.000-1.000]	0.799 [0.698-0.899]	0.502 [0.421-0.582]
**Ep. Data**[1]	0.668 [0.601-0.735]	0.968 [0.910-1.000]		0.786 [0.699-0.902]	0.501 [0.420-0.581]
**FT-IR**	0.676 [0.599-0.752]	0.966 [0.905-1.000]	1.000 [1.000-1.000]		0.538 [0.455-0.621]
**PFGE**	1.000 [1.000-1.000]	0.950 [0.899-1.000]	0.980 [0.967-0.993]	0.842 [0.767-0.918]	

95% CI, 95% confidence interval

[1] Ep. Data, Epidemiological Data. Hereby representing the frequency with which a given K-/KL-type has been described in the literature. In our collection, in case of multiple K-assignments, we considered the most frequent one and, in most cases, we confirmed it by WGS.
