# Supplementary material for: A Front Line on Klebsiella pneumoniae Capsular Polysaccharide Knowledge: Fourier Transform Infrared Spectroscopy as an Accurate and Fast Typing Tool
Source: mSystems. 2020 Mar 24;5(2):e00386-19. doi: 10.1128/mSystems.00386-19 (PMC7093823; doi:10.1128/mSystems.00386-19)
Supplement: TABLE S4 [file mSystems.00386-19-st004.pdf]

Table S4. Characteristics of the 154 international MDR *K. pneumoniae* clinical isolates analyzed in this study.

Strain	Origin	Year of isolation	Genotypic K-type/KL-type[a]	FT-IR K-type[b]	O-type[c]	ST/CG	PFGE Cluster	β-lactamases conferring resistance to extended-spectrum β-lactams
**C1733**	Portugal	2012	K19	1	O1	ST15/CG15	Kp1	CTX-M-15
**C1680**	Portugal	2012	K19	1	O1	ST15/CG15	Kp1	CTX-M-15
**C1694**[d]	Portugal	2012	K19	1	O1	ST15/CG15	Kp1	CTX-M-15
**K88**	Portugal	2015	K19	1	O1	ST15/CG15	Kp1	KPC-3
**K132**	Portugal	2015	K19	1	O1	ST15/CG15	Kp1	KPC-3
**K95**	Portugal	2015	K19	1	O1	ST15/CG15	Kp1	KPC-3
**4930/09**	Poland	2009	KL107	1	O2	ST258/CG258	Kp2	KPC-3
**2934/08**	Poland	2008	KL107	1	O2	ST258/CG258	Kp2	KPC-3, CTX-M-3
**12G9**	Spain	2012	KL151	1	O4	ST405/-	Kp3	OXA-48
**12G10**	Spain	2012	KL151	1	O4	ST405/-	Kp3	OXA-48
**12H41**	Spain	2012	KL151	1	O4	ST405/-	Kp3	OXA-48

Strain	Origin	Year of isolation	Genotypic K-type/KL-type[a]	FT-IR K-type[b]	O-type[c]	ST/CG	PFGE Cluster	β-lactamases conferring resistance to extended-spectrum β-lactams
**13I19**	Spain	2013	KL151	1	O4	ST405/-	Kp3	OXA-48
**C1702**	Portugal	2012	KL151	1	O4	ST405/-	Kp4	CTX-M-15
**H1144**	Portugal	2010	KL112[e]	1	O1	ST15/CG15	Kp5	CTX-M-15
**H1102**	Portugal	2010	KL112[e]	1	O1	ST15/CG15	Kp5	CTX-M-15
**H1099**	Portugal	2010	KL112[e]	1	O1	ST15/CG15	Kp5	CTX-M-15
**K8**	Portugal	2010	KL112[e]	1	O1	ST15/CG15	Kp5	CTX-M-15
**K9**	Portugal	2010	KL112[e]	2	O1	ST15/CG15	Kp5	CTX-M-15
**K10**	Portugal	2010	KL112[e]	1	O1	ST15/CG15	Kp5	CTX-M-15
**K2**	Portugal	2010	KL112[e]	1	O1	ST15/CG15	Kp5	CTX-M-15
**K23**	Portugal	2010	KL112[e]	1	O1	ST15/CG15	Kp5	CTX-M-15
**C1709**	Portugal	2012	KL112[e]	1	O1	ST15/CG15	Kp5	CTX-M-15
**H1185**	Portugal	2010	KL112[e]	1	O1	ST15/CG15	Kp5	CTX-M-15

Strain	Origin	Year of isolation	Genotypic K-type/KL-type[a]	FT-IR K-type[b]	O-type[c]	ST/CG	PFGE Cluster	β-lactamases conferring resistance to extended-spectrum β-lactams
**C1699**[d]	Portugal	2012	KL112[e]	1	O1	ST15/CG15	Kp5	CTX-M-15
**F12**	Brazil	2012	KL112[e]	1	O2	ST17/CG17	Kp6	SHV-2
**F29**	Brazil	2012	KL112[e]	1	O2	ST17/CG17	Kp6	SHV-2
**C1748**	Portugal	2012	KL112[e]	2	O5	ST17/CG17	Kp7	DHA-1, SHV-12
**18**	Romenia	2012	K17	2	O1	ST101/CG101	Kp8	OXA-48, CTX-M-15
**25**	Romenia	2012	K17	2	O1	ST101/CG101	Kp8	OXA-48, CTX-M-15
**35**	Romenia	2012	K17	2	O1	ST101/CG101	Kp8	OXA-48, CTX-M-15
**E45**	Romenia	2012	K17	1	O1	ST101/CG101	Kp8	OXA-48, CTX-M-15
**E7**	Romenia	2012	K17	1	O1	ST101/CG101	Kp8	OXA-48, CTX-M-15
**E16**	Romenia	2012	K17	1	O1	ST101/CG101	Kp8	OXA-181, NDM-1, CTX-M-15
**RP50**	Brazil	2012	K17	1	O1	ST101/CG101	Kp9	KPC-2, CTX-M-2
**RP73**	Brazil	2012	K17	1	O1	ST101/CG101	Kp9	KPC-2, CTX-M-2

**Strain**	**Origin**	**Year of isolation**	**Genotypic K-type/KL-type**[a]	**FT-IR K-type**[b]	**O-type**[c]	**ST/CG**	**PFGE Cluster**	**β-lactamases conferring resistance to extended-spectrum β-lactams**
**B23U**	Brazil	2012	K17	1	O1	ST101/CG101	Kp10	CTX-M-15
**B45U**	Brazil	2012	K17	1	O1	ST101/CG101	Kp10	CTX-M-15
**SC26**	Brazil	2012	K17	1	O1	ST101/CG101	Kp11	CTX-M-15
**H1120**	Portugal	2010	K24[f]	1	O1	ST15/CG15	Kp12	SHV-28
**H1098**	Portugal	2010	K24[f]	1	O1	ST15/CG15	Kp12	SHV-28
**H1100**	Portugal	2010	K24[f]	1	O1	ST15/CG15	Kp12	SHV-28
**Kp55**	Portugal	2014	K24[f]	1	O1	ST15/CG15	Kp12	OXA-48
**44**[d]	Portugal	2013	K24[f]	1	O1	ST15/CG15	Kp12	OXA-48, CTX-M-15
**C1686**	Portugal	2012	K24[f]	1	O1	ST15/CG15	Kp12	CTX-M-15
**C1713**	Portugal	2012	K24[f]	1	O1	ST15/CG15	Kp12	CTX-M-15
**C1693**	Portugal	2012	K24[f]	1	O1	ST15/CG15	Kp12	CTX-M-15
**C1700**	Portugal	2012	K24[f]	2	O1	ST15/CG15	Kp12	CTX-M-15

Strain	Origin	Year of isolation	Genotypic K-type/KL-type[a]	FT-IR K-type[b]	O-type[c]	ST/CG	PFGE Cluster	β-lactamases conferring resistance to extended-spectrum β-lactams
**H1119**[d]	Portugal	2010	K24[f]	2	O1	ST15/CG15	Kp12	SHV-2
**SC44**	Brazil	2012	K24[f]	1	O1	ST15/CG15	Kp12	CTX-M-15
**K21**	Portugal	2010	K24[f]	1	O1	ST15/CG15	Kp12	CTX-M-15
**H693**	Portugal	2006	K24[f]	1	O1	ST15/CG15	Kp12	CTX-M-15
**H646**	Portugal	2006	K24[f]	2	O1	ST11/CG258	Kp13	DHA-1
**13I15**	Spain	2012	K24[f]	1	O2	ST11/CG258	Kp14	OXA-48
**12E76**	Spain	2012	K24[f]	1	O2	ST11/CG258	Kp14	OXA-48
**12F14**	Spain	2012	K24[f]	1	O2	ST11/CG258	Kp14	OXA-48
**12F48**	Spain	2012	K24[f]	1	O2	ST11/CG258	Kp14	OXA-48
**12F64**	Spain	2012	K24[f]	1	O2	ST11/CG258	Kp14	OXA-48
**12F72**	Spain	2012	K24[f]	1	O2	ST11/CG258	Kp14	OXA-48
**12F73**	Spain	2012	K24[f]	1	O2	ST11/CG258	Kp14	OXA-48

**Strain**	**Origin**	**Year of isolation**	**Genotypic K-type/KL-type**[a]	**FT-IR K-type**[b]	**O-type**[c]	**ST/CG**	**PFGE Cluster**	**β-lactamases conferring resistance to extended-spectrum β-lactams**
**12F55**	Spain	2012	K24[f]	1	O2	ST11/CG258	Kp14	OXA-48
**12G17**	Spain	2012	K24[f]	1	O2	ST11/CG258	Kp14	OXA-48
**12H72**	Spain	2012	K24[f]	1	O2	ST11/CG258	Kp14	OXA-48
**10E34**[d]	Spain	2010	K24[f]	1	O2	ST11/CG258	Kp15	KPC-3
**H1157**	Portugal	2010	K16[f]	1	O1	ST14/CG14	Kp16	SHV-106
**H1122**[d]	Portugal	2010	K16[f]	1	O1	ST14/CG14	Kp16	SHV-106
**H1096**	Portugal	2010	K16[f]	1	O1	ST14/CG14	Kp16	SHV-106
**H1188**	Portugal	2010	K16[f]	1	O1	ST14/CG14	Kp16	SHV-55
**H1011**	Portugal	2010	K16[f]	1	O1	ST14/CG14	Kp16	SHV-55
**H39**	Portugal	2003	K16[f]	1	O1	ST14/CG14	Kp16	SHV-55
**RP29**	Brazil	2012	K27	1	O2	ST11/CG258	Kp17	KPC-2, CTX-M-2
**RP66**	Brazil	2012	K27	1	O2	ST11/CG258	Kp17	KPC-2, CTX-M-2

Strain	Origin	Year of isolation	Genotypic K-type/KL-type[a]	FT-IR K-type[b]	O-type[c]	ST/CG	PFGE Cluster	β-lactamases conferring resistance to extended-spectrum β-lactams
**RP75**	Brazil	2012	K27	1	O2	ST11/CG258	Kp17	CTX-M-2
**RP65**	Brazil	2012	K27	1	O2	ST11/CG258	Kp17	CTX-M-2
**RP52**	Brazil	2012	K27	1	O2	ST11/CG258	Kp17	CTX-M-2
**RP80**	Brazil	2012	K27	1	O2	ST11/CG258	Kp17	CTX-M-2
**RP28**	Brazil	2012	K27	1	O2	ST11/CG258	Kp18	CTX-M-2
**RP82**	Brazil	2012	K27	1	O2	ST11/CG258	Kp19	CTX-M-2
**09B76**	Spain	2009	K60	1	O1	ST253/-	Kp20	VIM-1
**09C12**	Spain	2009	K60	1	O1	ST253/-	Kp20	VIM-1
**10F74**	Spain	2010	K60	1	O1	ST253/-	Kp20	VIM-1
**K43**	Portugal	2011	KL110	1	O1	ST15/CG15	Kp21	VIM-34, SHV-12, OXA-17
**K47**	Portugal	2012	KL110	1	O1	ST15/CG15	Kp21	VIM-34, SHV-12, OXA-17
**C1682**	Portugal	2012	K62	1	O1	ST348/-	Kp22	CTX-M-15

**Strain**	**Origin**	**Year of isolation**	**Genotypic K-type/KL-type**[a]	**FT-IR K-type**[b]	**O-type**[c]	**ST/CG**	**PFGE Cluster**	**β-lactamases conferring resistance to extended-spectrum β-lactams**
**C1685**	Portugal	2012	K62	1	O1	ST348/-	Kp22	CTX-M-15
**C1741**	Portugal	2012	K62	1	O1	ST348/-	Kp22	CTX-M-15
**Kp56**	Portugal	2014	K62	1	O1	ST348/-	Kp22	KPC-3, CTX-M-15
**H1170**	Portugal	2010	- (*wzi*150)[g]	1	-	ST336/CG17	Kp23	CTX-M-15
**H1160**	Portugal	2010	- (*wzi*150)[g]	1	-	ST336/CG17	Kp23	CTX-M-15
**H1182**	Portugal	2010	- (*wzi*150)[g]	1	-	ST336/CG17	Kp23	CTX-M-15
**H1162**	Portugal	2010	- (*wzi*150)[g]	1	-	ST336/CG17	Kp23	CTX-M-15
**H1156**	Portugal	2010	- (*wzi*150)[g]	1	-	ST336/CG17	Kp23	CTX-M-15
**H1148**	Portugal	2010	- (*wzi*150)[g]	1	-	ST336/CG17	Kp23	CTX-M-15
**H1139**	Portugal	2010	- (*wzi*150)[g]	1	-	ST336/CG17	Kp23	CTX-M-15
**H1128**	Portugal	2010	- (*wzi*150)[g]	1	-	ST336/CG17	Kp23	CTX-M-15
**H1113**	Portugal	2010	- (*wzi*150)[g]	1	-	ST336/CG17	Kp23	CTX-M-15

Strain	Origin	Year of isolation	Genotypic K-type/KL-type[a]	FT-IR K-type[b]	O-type[c]	ST/CG	PFGE Cluster	β-lactamases conferring resistance to extended-spectrum β-lactams
**H1101**	Portugal	2010	- (*wzi*150)[g]	1	-	ST336/CG17	Kp23	CTX-M-15
**H1097**	Portugal	2010	- (*wzi*150)[g]	1	-	ST336/CG17	Kp23	CTX-M-15
**H1118**	Portugal	2010	- (*wzi*150)[g]	1	-	ST336/CG17	Kp23	CTX-M-15
**CRE01 KP**	Brazil	2008	KL106[e]	1	O2	ST258/CG258	Kp24	KPC-2
**HCC52**	Brazil	2007	KL106[e]	1	O2	ST258/CG258	Kp24	KPC-2
**HCC02**	Brazil	2007	KL106[e]	1	O2	ST258/CG258	Kp24	KPC-2
**CRE38**	Brazil	2006	KL106[e]	1	O2	ST258/CG258	Kp24	KPC-2
**HCC93**	Brazil	2009	KL106[e]	1	O2	ST258/CG258	Kp24	KPC-2
**HCC49**	Brazil	2009	KL106[e]	1	O2	ST258/CG258	Kp24	KPC-2
**126/09**	Poland	2008	KL106[e]	1	O2	ST258/CG258	Kp2	KPC-2, SHV-12
**5023/09**	Poland	2009	KL106[e]	1	O2	ST258/CG258	Kp2	KPC-2, CTX-M-3
**5586/09**	Poland	2009	KL106[e]	1	O2	ST258/CG258	Kp2	KPC-2, CTX-M-3, SHV-12

**Strain**	**Origin**	**Year of isolation**	**Genotypic K-type/KL-type**[a]	**FT-IR K-type**[b]	**O-type**[c]	**ST/CG**	**PFGE Cluster**	**β-lactamases conferring resistance to extended-spectrum β-lactams**
**6595/09**	Poland	2009	KL106[e]	1	O2	ST258/CG258	Kp2	KPC-2, SHV-12
**Kp4077**	Greece	2007	KL106[e]	1	O2	ST258/CG258	Kp25	KPC-2
**Kp1810**	Greece	2007	KL106[e]	1	O2	ST258/CG258	Kp25	KPC-2
**Kp1664**	Greece	2007	KL106[e]	2	O2	ST258/CG258	Kp25	KPC-2
**Kp1652**	Greece	2007	KL106[e]	2	O2	ST258/CG258	Kp25	KPC-2
**H677**	Portugal	2006	K64[h]	1	O2	ST147/CG147	Kp26	SHV-12
**H1168**	Portugal	2010	K64[h]	1	O2	ST147/CG147	Kp26	SHV-12
**H1183**	Portugal	2010	K64[h]	1	O2	ST147/CG147	Kp26	SHV-12
**H1134**	Portugal	2010	K64[h]	1	O2	ST147/CG147	Kp26	SHV-12
**H1143**	Portugal	2010	K64[h]	1	O2	ST147/CG147	Kp26	SHV-12
**H1110**	Portugal	2010	K64[h]	1	O2	ST147/CG147	Kp26	SHV-12
**H1108**	Portugal	2010	K64[h]	1	O2	ST147/CG147	Kp26	SHV-12

**Strain**	**Origin**	**Year of isolation**	**Genotypic K-type/KL-type**[a]	**FT-IR K-type**[b]	**O-type**[c]	**ST/CG**	**PFGE Cluster**	**β-lactamases conferring resistance to extended-spectrum β-lactams**
**K70**	Portugal	2015	K64[h]	1	O2	ST147/CG147	Kp26	KPC-3, SHV-12
**K89**	Portugal	2015	K64[h]	1	O2	ST147/CG147	Kp26	KPC-3
**K93**	Portugal	2015	K64[h]	1	O2	ST147/CG147	Kp26	KPC-3
**K74**	Portugal	2015	K64[h]	1	O2	ST147/CG147	Kp26	KPC-3
**K126**	Portugal	2015	K64[h]	1	O2	ST147/CG147	Kp26	KPC-3
**K105**	Portugal	2015	K64[h]	1	O2	ST147/CG147	Kp26	KPC-3
**K112**	Portugal	2015	K64[h]	1	O2	ST147/CG147	Kp26	KPC-3
**10D79**	Spain	2010	K64[h]	1	O1	ST147/CG147	Kp27	VIM-1
**RP32**	Brazil	2012	K64[h]	1	O2	ST11/CG258	Kp28	CTX-M-2
**B40U**	Brazil	2012	K64[h]	1	O2	ST11/CG258	Kp28	CTX-M-2
**B31U**	Brazil	2012	K64[h]	1	O2	ST11/CG258	Kp28	KPC-2, CTX-M-2
**09B51**	Spain	2009	K14	1	O3	ST54/-	Kp29	VIM-1

Strain	Origin	Year of isolation	Genotypic K-type/KL-type[a]	FT-IR K-type[b]	O-type[c]	ST/CG	PFGE Cluster	β-lactamases conferring resistance to extended-spectrum β-lactams
**10H66**	Spain	2010	K14	1	O3	ST54/-	Kp29	VIM-1
**161**	Spain	2010	K14	1	O3	ST54/-	Kp29	VIM-1
**H642**[d]	Portugal	2006	KL105	1	O2	ST11/CG258	Kp30	DHA-1
**H830**	Portugal	2008	KL105	1	O2	ST11/CG258	Kp30	DHA-1
**H688**	Portugal	2007	KL105	1	O2	ST11/CG258	Kp30	DHA-1
**C1721**	Portugal	2012	KL105	1	O2	ST11/CG258	Kp30	DHA-1
**H2076**	Portugal	2012	KL105	1	O2	ST11/CG258	Kp30	DHA-1
**H1523**[d]	Portugal	2011	KL105	2	O2	ST11/CG258	Kp30	DHA-1
**C1951**	Portugal	2013	KL105	2	O2	ST11/CG258	Kp30	DHA-6
**HCC23**	Brazil	2009	KL127[f]	1	-	ST11/CG258	Kp31	KPC-2
**SC51**	Brazil	2012	KL127[f]	1	-	ST11/CG258	Kp31	KPC-2
**H49**[d]	Portugal	2003	K2[f]	1	O1	ST14/CG14	Kp32	TEM-24

Strain	Origin	Year of isolation	Genotypic K-type/KL-type[a]	FT-IR K-type[b]	O-type[c]	ST/CG	PFGE Cluster	β-lactamases conferring resistance to extended-spectrum β-lactams
**H55**	Portugal	2002	K2[f]	1	O1	ST14/CG14	Kp32	TEM-24
**H153**	Portugal	2003	K2[f]	1	O1	ST14/CG14	Kp32	TEM-24
**08Z37**	Spain	2008	K23	1	O1	ST39/CG39	Kp33	VIM-1
**Kpn20**	Spain	2008	K23	1	O1	ST39/CG39	Kp33	VIM-1
**09C77**	Spain	2009	K23	1	O1	ST39/CG39	Kp33	VIM-1
**09A69**	Spain	2009	K23	1	O1	ST39/CG39	Kp33	VIM-1
**10D60**	Spain	2010	K23	1	O1	ST39/CG39	Kp33	VIM-1
**10F53**	Spain	2010	K23	1	O1	ST39/CG39	Kp33	VIM-1
**160**	Spain	2010	K23	1	O1	ST39/CG39	Kp33	VIM-1
**H1111**	Portugal	2010	KL48	NI	O1	ST15/CG15	Kp12	SHV-12
**09B53**	Spain	2012	- (*wzi*200)[g]	NI	-	ST17/CG17	Kp34	VIM-1

-, not defined; NI, not included

[a] Capsular (K)-type defined according to Brisse *et al*. [14] and capsular locus (KL)-type according to Wick *et al.* [7] (http://bigsdb.pasteur.fr/perl/bigsdb/bigsdb.pl?db=pubmlst_klebsiella_seqdef_public&page=sequenceQuery).
[b] 1, predicted; 2, exception.
[c] O-types defined according to Fang *et al*.[10].
[d] Isolates selected for whole-genome sequencing (WGS).
[e] K locus (KL) genetically different from the K-type positive reaction reported in Brisse *et al*.[14].
[f] Different KL types reported for these *wzi* alleles – the KL-type was assumed according to WGS results (K24, K16, KL2) or by frequency distribution (KL127).
[g] *wzi* allele associated with none or multiple K/KL-types.
[h] Cross reaction between K14 and K64, solved by sequencing of *wzy*
